# Depression in Parkinson's Disease: A Narrative Review

**DOI:** 10.7759/cureus.27750

**Published:** 2022-08-07

**Authors:** Rahul Chikatimalla, Thejaswi Dasaradhan, Jancy Koneti, Swathi Priya Cherukuri, Revanth Kalluru, Sai Gadde

**Affiliations:** 1 Research, Kamineni Institute of Medical Sciences, Narketpally, IND; 2 Internal Medicine, Narayana Medical College, Nellore, IND; 3 Internal Medicine, Kamineni Institute of Medical Sciences, Narketpally, IND; 4 Research, Narayana Medical College, Nellore, IND

**Keywords:** monoamine oxidase theory, selective serotonin reuptake inhibitor (ssri), electroconvulsive therapy (ect), mao inhibitors, neuroinflammation, depression in parkinson's disease, depression, parkinson's disease

## Abstract

Parkinson's disease (PD) is a progressive neurodegenerative age-related disorder that affects the central nervous system (CNS) and is characterized by uncontrollable movements such as shaking, stiffness, and loss of balance and coordination. Depression is a common non-motor manifestation of PD, but unfortunately, depression remains unrecognized and often undertreated. The underlying pathophysiology of depression in PD is complicated, and many studies have been conducted to know the exact cause, but the question remains unanswered.

In this article, we discuss various pathophysiologies by which depression occurs in PD. The most widely accepted theories are neuroinflammation and monoamine oxidase theory. This article also explored the pharmacological treatment of depression in PD; this involves standard antidepressant therapy such as tricyclic antidepressants (TCA), serotonin-norepinephrine reuptake inhibitors (SNRI), selective serotonin reuptake inhibitors (SSRI), and monoamine oxidase inhibitors (MAO); non-pharmacological treatments such as electroconvulsive therapy (ECT), cognitive-behavioral therapy (CBT) have also been discussed. However, physicians hesitate to prescribe antidepressants to patients with PD due to concerns about harmful drug-drug interactions between antidepressants and antiparkinsonian drugs. Despite the complicated link between PD and depression, the co-administration of antidepressants and antiparkinsonian drugs is safe and beneficial when appropriately managed. However, early recognition and initiation of treatment of depression in PD reduces the longitudinal course and improves the cross-sectional picture. This review article also explored the clinical and diagnostic findings and impact on the quality of life of depression in PD.

## Introduction and background

Parkinson's disease (PD) is a progressive neurodegenerative age-related disorder with rigidity, bradykinesia, postural instability, and tremors. It is usually manifested with both motor and non-motor symptoms [1]. The prevalence of PD in those who are aged 45 years and above is estimated to be 572 per 100,000 (95% confidence interval (CI) 537-614), based on the US demographic structure from 2010. A total of 680,000 people aged ≥ 45 years are affected with PD in the US in 2010, and according to forecasts from the US Census Bureau population projections, that figure will increase to 930,000 in 2020 and 1,238,000 in 2030 [2]. Significant implications for brain-related disease risk and sex differences in brain structure and function depend on fetal hormonal programming and sex-determining genes. The biological differences in the expression of neurodegenerative diseases such as PD depend on the factors mentioned above, along with physical changes concerning age [3]. Following the survey conducted by the Global Burden of Disease, Injuries, and Risk Factors Study (GBD), the number of people living with PD has risen drastically from 2.5 million to 6.1 million within 26 years. The most important risk factors are industrial chemicals, advanced age, and pollution. Smoking, coffee, and a few others function as protective factors [4].

PD manifests as a Mendelian type with autosomal dominant or recessive inheritance in 5%-10% of patients. Until the early 1990s, the genetic contribution to PD was dismissed; nevertheless, 15 years after the first gene discovered was linked to an autosomal dominant type of the disease, we now know of 28 unique chromosomal regions linked to the disease [5]. α-synuclein (SNCA) and leucine-rich repeat kinase 2 (LRRK2) for autosomal dominant PD, and PINK1, PARK7 (DJ-1), ATPase type 13A2 (ATP13A2), and PARK2 (parkin) for autosomal recessive PD, have been identified as the underlying genes that generate common monogenic types of PD in only six of these locations [6]. Typical problems of PD patients, including depigmentation, neuronal death, and gliosis, affect the substantia nigra pars compacta and the pontine locus coeruleus from a pathogenic standpoint. About 60%-70% of the neurons in the substantia nigra will be affected by the time the PD symptoms appear [7,8]. The major burden of pathology in a particular brain region is represented by abnormal alpha-synuclein with neurites, but in some regions like the basal ganglia, the most detected abnormality is alpha-synuclein with or without the presence of Lewy bodies. In most cases, oligodendroglial inclusions within the midbrain and basal ganglia can be seen, in which most of the alpha-synuclein pathology is within the neurons [9].

Depression is one of the most important determinants of poorer quality of life in PD patients. They lead to poor cognitive performance, lower quality of life, a worse functional status, and an increased risk of death. Early detection and diagnosis are critical since depression significantly impacts the quality of life of patients and caregivers; effective treatment can alleviate these symptoms, thus the outcome and prognosis of PD patients [10]. This review article aims to highlight the depression pathophysiology in people with PD, explore the clinical interrelation between PD and depression, and highlight the diagnostic and therapeutic possibilities.

## Review

Although no mechanism explaining the exact linkage between depression and PD has been identified yet, several mechanisms that co-exist between them have been implicated. The loss in cognition and other frequent symptoms of both these diseases are caused by several mechanisms [11]. When PD patients are matched for impairment with patients with other chronic debilitating diseases, neurobiological variables linked with the underlying neurodegenerative disease and its somatic therapies create a backdrop for the greater prevalence of depressive symptoms rather than psychosocial factors [12]. The development and natural history of depressive syndromes differ from that of motor disturbances. Depression is two times more likely in the "premotor" years preceding PD diagnosis, indicating that the neurodegenerative process has a role in prodromal mood disorders as the movement disease develops [13]. Monoaminergic abnormalities, Lewy body pathology, functional changes in limbic and subcortical circuits, hippocampal atrophy, variations in neurogenesis and neurotrophic factors, and toxic stress with hypercortisolemia and inflammation, are all thought to have a role in late-life depression [14].

Genetic factors and pathophysiology

Non-motor symptoms have not been routinely evaluated in investigations of genetic variables in PD. None of the genetic variants related to PD or depressive disorders have been identified to be connected specifically with PD depression [15].

Depression and other neuropsychological abnormalities are more frequent in PD patients with LRRK2 G2019S mutation. Since a single G2019S mutation produces a major proportion of autosomal dominant types of PD, the LRRK2 gene, which encodes dardarin, changed the genetics of PD. As a result, it is the most detected mutation in PD discovered to date, as per the study done by Belarbi et al., which was published in 2010 and was performed at a clinic in Algeria. This study included 106 patients, of which 23 were G2019S mutation carriers and 48 noncarriers; both the patient groups had similar ages at onset and included both male and female patients. The clinical symptoms of PD in G2019S mutant carriers and noncarriers were identical in this investigation, except for L-Dopa-induced dyskinesias, which were substantially more common in the G2019S mutation group (53%) than in the noncarrier group (16%). This subset of PD patients was not evaluated for non-motor symptoms. G2019S mutation carriers were 23 (11 males, 12 females), and noncarriers were 48 (34 males, 14 females) of the 71 patients (45 males, 26 females) who underwent cognitive and neuropsychiatric tests. The two groups of patients were of similar ages at the time of onset and evaluation. This study concluded that there is greater involvement of the limbic system in patients with LRRK2 G2019S mutation, and depression and hallucinations are more frequent among them [16].

In a separate study among PD patients, compared to relatives of probands without PARK2 mutations, unaffected relatives of probands with compound heterozygous PARK2 mutations had greater depression symptom scores. Srivastava et al. performed a study over five years at Columbia University in New York, which was published in 2012. The study comprised 328 patients (88 probands and 240 relatives), all of whom had genotyping and psychological data. There were 218 first-degree relatives (40 parents, 100 siblings, and 78 children) and 22 second-degree relatives among the 240 relatives. When compared to the other two groups, compound heterozygotes (n=16) and homozygotes (n=4) had a considerably younger age at onset and significantly longer disease duration (heterozygotes and subjects with no mutations) [17]. This study concluded that when compared to relatives without parkin mutations, relatives of early-onset PD individuals with compound heterozygous mutations and no confirmed PD may have a greater risk of depression.

According to a recent study in PD patients, a functional polymorphism in the promoter of the 5HTT gene (5HTT gene-linked polymorphic region, 5 HTTLPR) determines high or low 5HT uptake and is connected to depression symptoms [18].

Neurodegeneration and Depression Linkage

Although the definitive mechanism by which depression in PD occurs has not been discovered yet, many theories have been postulated. the monoamine oxidase theory is the most widely accepted in depressive disease pathology. Another most intensely researched mechanism is inflammation in the central nervous system (CNS) and its linkage to neuropsychiatry. Several other pathways are thought to be involved, but untill now, the exact mechanism is unknown [11]. Degeneration of monoaminergic neurotransmitter systems and fronto-cortical dysfunctions appear to be the root causes of depression in PD. Depressed PD patients had substantial cell death in the nucleus coeruleus, the primary source of brain noradrenaline, according to postmortem examinations. In depressive PD patients, morphological changes in the nucleus raphe, which is the principal source of brain serotonin, have been observed [19]. Dopaminergic mesocortical and mesolimbic neuron degeneration causes orbitofrontal cortex dysfunction, which affects serotonergic cell bodies in the dorsal raphe nuclei secondarily. The orbitofrontal cortex-basal ganglia-thalamic circuit, which connects the orbitofrontal cortex to the anterior temporal cortex via the uncinate fasciculus, and the orbitofrontal cortex-basal ganglia-thalamic circuit are the two other circuits that have been proposed to be affected in patients with depression [20].

Neuroinflammation

**Figure 1:**
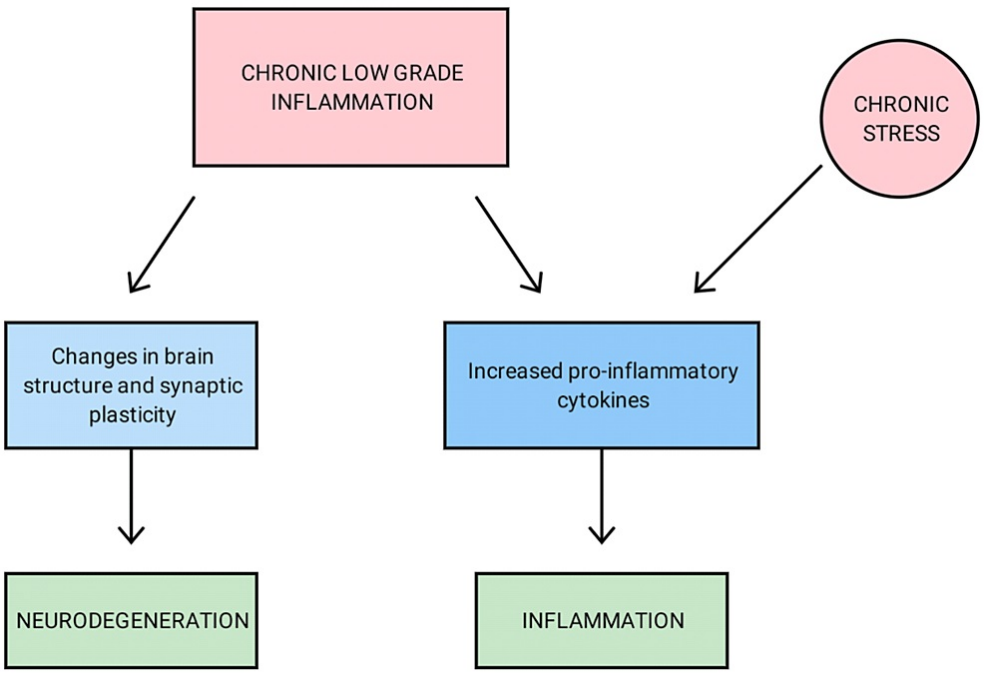
Pathogenesis of neurodegeneration due to chronic low-grade Inflammation Image credits - Rahul Chikatimalla

The brain's response to injury, infection, or disease is known as neuroinflammation. Inflammation's overall goal is to eliminate or inactivate potentially harmful substances or damaged tissue. Glia in the CNS and lymphocytes, monocytes, and macrophages in the hematopoietic system are the primary mediators of this reaction [21,22]. Microglial cells of the CNS play a significant role in maintaining the immune response of the brain [23]. Any local insult or systemic insult can lead to microglial cell activation, which induces the release of both proinflammatory and anti-inflammatory cytokines in balance. If there is chronic low-grade inflammation, it could lead to exacerbating the release of proinflammatory cytokines such as tumor necrosis factor alpha (TNF-alpha), interferon gamma (IFN-gamma), IL-1, and IL-6 (Figure 1) [24]. Chronic low-grade inflammation has been linked to alterations in brain structure and synaptic plasticity, which could lead to neurodegeneration [25-28]. The release of both pro-and anti-inflammatory cytokines can occur shortly after glial cell activation. The balance between these conflicting responses determines the cytokine's final effect.

**Figure 2:**
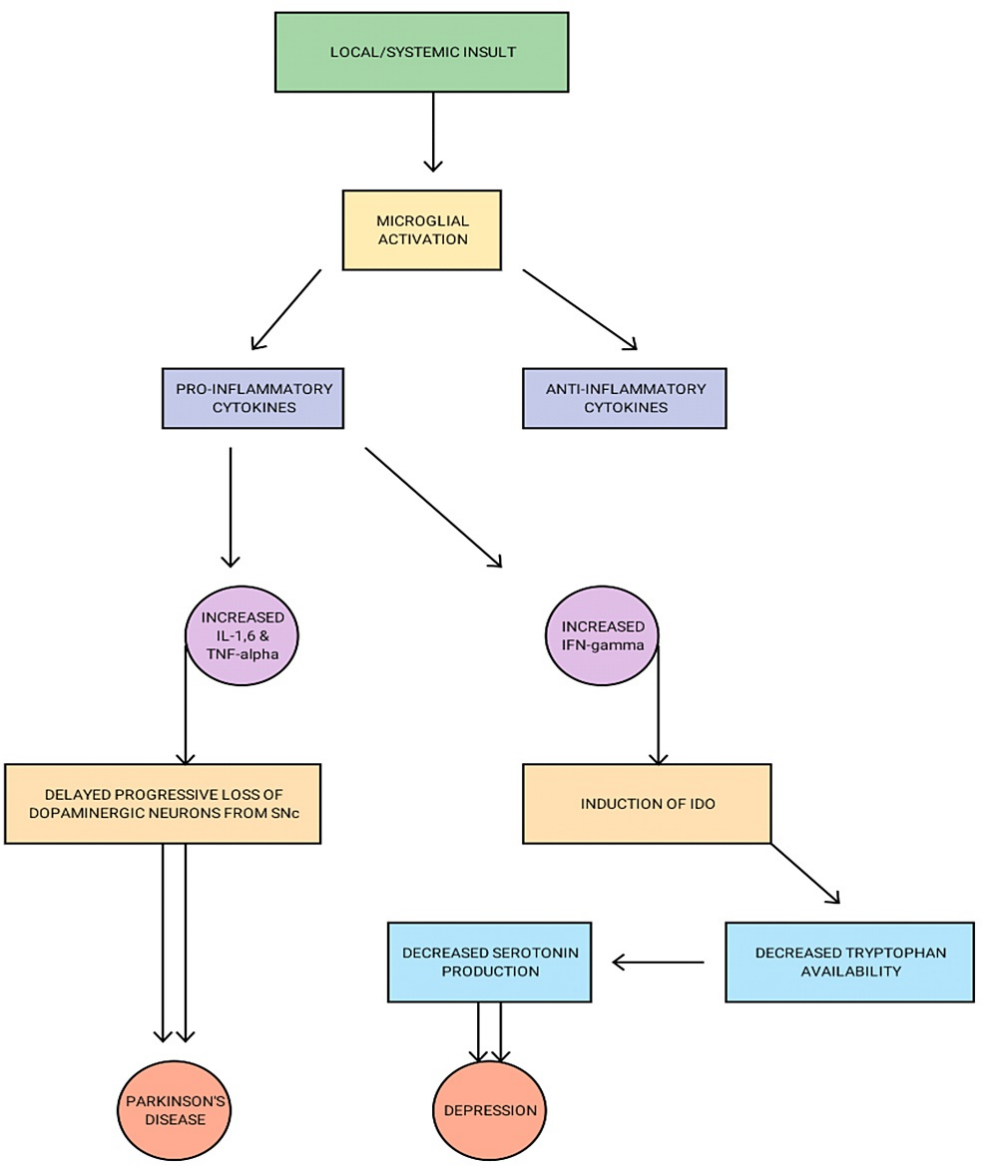
Pathogenesis of depression and Parkinson's disease due to neuroinflammation IL - Interleukin, TNF - Tumor necrosis factor, IFN - Interferon, SNc - Substantia nigra, IDO - Indoleamine-pyrrole 2,3-dioxygenase Image credits - Rahul Chikatimalla

In this vein, it is hypothesized that glial activation has a protective effect at first, but that continued stimulation can result in excessive proinflammatory cytokine production and cause neuronal injury [29]. Lipopolysaccharide (LPS)-induced inflammation, which mimics disease-induced inflammation, induces a long-term rise in TNF-alpha from the brain microglia months after the inflammation has been abated in the periphery. The increased proinflammatory response causes a delayed and progressive loss of dopaminergic neurons in the substantia nigra, comparable to PD, showing that uncontrolled neuroinflammation may lead to neurodegeneration [30]. There is a significant reduction in tryptophan availability due to an enzyme called indoleamine 2,3- dioxygenase (IDO), which can be induced by IFN-gamma, leading to decreased synthesis of serotonin in the brain (Figure 2). Because serotonin has been linked to depression and neurodegeneration, there is even more evidence that an uncontrolled inflammatory response can play a role in these conditions [31].

Many investigations have found that proinflammatory cytokines such as interleukin-6 are elevated in the cerebrospinal fluid of patients with neurodegenerative disorders and depression. Neurotrophic factors like BDNF, as well as anti-inflammatory cytokines like IL-10, are reduced [32].

Monoamine Oxidase Theory

According to the monoamine oxidase theory, PD patients most likely experience depression that usually results from neurodegeneration of dopaminergic, cholinergic, serotonergic, and nor-adrenergic neurons [33-35]. In PD, free iron accumulates inside some melanin-containing dopaminergic neurons, as well as inside amyloid plaques and neurofibrillary tangles linked to PD dementia [36,37]. The iron buildup has been hypothesized as a contributor to the oxidative stress-induced apoptosis seen in both PD and PD dementia [37,38]. Increased glial monoamine oxidase inhibitors (MAO) activity can lead to increased hydrogen peroxide generation, which can generate reactive hydroxyl radicals via Fenton chemistry with intracellular ferrous iron [39,40]. Selective MAO inhibitors are also being researched for their potential application in depression and neurodegenerative disorders.

Therefore, these complicated interactions between chronic stress, neuroinflammation, and monoamine pathways, along with various other factors, lead to increased production of proinflammatory cytokines, increased free radicals, and decreased neuroprotectants, predisposing patients to both neurodegenerative and psychiatric disorders. Despite many theories, literature still lacks the exact interactions linked to all these pathways that explains these complex manifestations.

Epidemiology and clinical presentation and impact on quality of life

Epidemiology of Depression in PD

The prevalence of depression in PD patients ranges from 2.7% to 90%, depending on the study [41]. According to a meta-analysis of 15 studies from 10 countries, the prevalence of major depressive disorder in people with PD was 22.9% (95% CI 18.1-27.7) [42]. In a cohort study of newly diagnosed untreated PD patients, 13.9% experienced depression at the start, which was nearly double the healthy control group [43]. Depressive symptoms are thought to affect 40%-50% of people with PD. Depression is the most common neuropsychiatric disorder among people with PD. The claimed prevalence, on the other hand, varies depending on the definitions of "case-ness," the population sampled, the depression subtypes or discrete mood disorders evaluated, and the presentation and coarse heterogeneity. Select cutoff scores on self-report or clinician-rated psychiatric symptom rating measures can also be used to determine case-ness [41]. Schuurman et al. found a hazard ratio of 3.13 (95% CI 1.95-5.01) for individuals with depression to acquire PD when compared to those who did not have depression in a retrospective cohort study [44]. In a seven-year analysis of 353 individuals, those diagnosed with depression after PD were more likely to acquire dementia than those diagnosed with depression before PD (HR = 2.01 [95% CI 1.14-3.53]) [45].

The extent of depressive symptoms is mild to severe in most PD samples. Rojo et al. conducted a prospective cohort study that was performed over a period of nine years at a neurology service hospital in Barcelona which was published in 2003. This study included 353 patients, of which 140 were male patients and 208 were female patients, and additional follow-up information was obtained for 184 patients [46]. This study concluded that a substantial number of people with PD experience moderate to severe depression symptoms. Anxiety disorders, cognitive impairment, and psychosis are all more likely to be accompanied by depressive symptoms.

The most powerful predictor of beginning dopaminergic treatment was depressive symptoms rather than motor impairments. Ravina et al. conducted a double-blinded randomized controlled trial at NIH over a period of 12-18 months which was published in 2009. This study included 413 patients with recently diagnosed but untreated PD, of which 114 (27.4%) screened positive for clinically significant depressive symptoms over a 15-month follow-up period. While mild depressed symptoms at the baseline were associated with a sixfold increased risk of developing moderate to severe depression symptoms over the follow-up period, depressive symptoms disappeared in half of the patients with clinically relevant mild symptoms by six months. This study concluded that depressive symptoms play a significant role in disability and the choice to begin symptomatic therapy for motor-related impairment in early PD, emphasizing the necessity of recognizing and treating depression in this population [47].

Diagnosis and Clinical Findings

With PD or any other neurodegenerative illness, psychological disorders, notably depression, are common. Due to the overlap of symptoms between depression and these conditions, it can be difficult to diagnose because depression can also present with neurological symptoms, including cognitive impairment [48]. Situational affective disorders, such as emotional reactions to specific worries or circumstances, grief, and adjustment disorders, are often not classified as affective disorders because they resolve when the individual adapts to the stressor. A clinically depressed state can include an inability to adjust to a stressor, whether caused by a PD event or something else [49].

One of the most prevalent psychological symptoms among dementia patients is irritability. Verkaik et al. performed a cross-sectional study in psychogeriatric wards of nursing homes in Dutch on 518 patients with dementia, which was published in 2009. This study was performed in the Netherlands in about 27 nursing homes with inhabitants of 1000 beds who were 65 years of age and above [50]. This study concluded that in psychogeriatric nursing homewards, the prevalence of comorbid depression in dementia (stages 2-6) was 19%, and the most seen depressive symptoms were irritability and fatigue. The average number of depressed symptoms was 5.6 (SD 1.84), with no significant differences between dementia stages. As a result, irritability should alert caregivers to the presence of depression and may aid in early detection.

The primary subtypes of depression diagnoses in people with PD (major depression, moderate depression, and dysthymia) are like depressive subtypes in non-PD people. Depressive disorders are characterized by repeated changes in mood, including the ability to experience pleasure (hedonic ability), as well as pronounced somatic signs and complaints, cognitive changes, and vegetative symptoms (sleep and appetite disturbances) that overlap with the characteristics of PD [49].

Impact on Quality of Life

Increased disability, a poorer health-related quality of life (HRQOL), accelerated progression of motor impairment/disability, and increased mortality has all been linked with depression [51]. In the preliminary stages of the disease, psychiatric-related symptoms have a stronger influence on HRQOL than in the later stages. This suggests that psychological symptoms like anxiety and depression are not only a reaction to the stress of living with a long-term debilitating neurological condition but are an essential element of the neuropathological process [52].

Menon et al. performed a study in a PD clinic in a tertiary referral hospital, which was published in 2015. This study included 65 patients diagnosed with idiopathic PD composed of 20 female patients and 45 male patients with a mean age group of 60. This study concluded that the presence of depression is the most important predictor of low quality of life, and not the severity of motor symptoms or the length of the illness. HRQOL is the essential metric of a patient's handicap and a reflection of the felt disability from the patient's perspective. Non-motor symptoms in PD should be evaluated and treated early on to enhance HRQOL and patient productivity, reduce morbidity, and lower direct and indirect healthcare costs [52].

Unfortunately, there are no diagnostic tests or biochemical markers for determining whether someone is depressed [53]. When there is no other way to diagnose it, clinicians must rely on the patient's history and numerous diagnostic scales and criteria. These tools are insufficient to rely on when it occurs with other neurological diseases. As a result, molecular indicators, as well as stronger and more clear clinical criteria and a high degree of suspicion for depression in older adults with neurodegenerative disease, are needed [54].

Treatment modalities

When depressive symptoms continue and cause distress or functioning, treatment is recommended. Treatment should be modulated to the patient's needs and multifaceted, including medications as needed, education about mood disorders and how they relate to PD, encouragement of skills to enhance mood symptom coping methods, and emotional support [49]. Assessment of motor fluctuations and ensuring optimal treatment of motor symptoms should be the first step in treating depression in people with PD. Because of the link between 'off-periods' and depressive symptoms, optimizing dopaminergic medication is critical [55]. A recent meta-analysis evaluated 13 trials that focused on pharmacologic interventions for depression in people with PD [56]. Most of these trials focused on the use of selective serotonin reuptake inhibitors (SSRI) [57-59] and tricyclic antidepressants (TCA) [57,59,60]. Some of the trials also reviewed the use of dopamine agonists and non-standard depression agents, trazodone, memantine (MEM), and atomoxetine. The meta-analysis concluded that pharmacologic intervention with standard antidepressant drugs had a substantial aggregate effect on the treatment of depression in PD; however, further stratification revealed that the effect was significant only within the SSRI group [57].

Pharmacological Treatment

Depression that occurs in conjunction with neurodegenerative disorders such as PD has been demonstrated in studies to be largely or completely unresponsive to typical antidepressant therapies. Traditional antidepressants such as SSRI and serotonin-norepinephrine reuptake inhibitors (SNRI), in combination with other drugs such as cholinesterase inhibitors and MAO inhibitors, are currently used to treat depression in patients with neurodegenerative diseases [61,62].

SSRI*:* SSRIs are thought to help with depression by preventing serotonin (5-HT) from being reabsorbed and therefore, increases its availability in the synaptic cleft. SSRIs are commonly used to treat individuals with depression, regardless of a concomitant diagnosis of PD, because of their relative tolerance and safety in overdose [61]. SSRI drugs such as citalopram, sertraline, paroxetine, and fluoxetine have been studied in several randomized controlled trials (RCTs) for the treatment of PD [57-59]. Three of these trials found that patients with PD had improved their standardized depression scores; however, only very few showed complete remission of depressive symptoms. Some case reports and trial findings imply that SSRIs may exacerbate PD's motor symptoms [63]. Patients with PD are frequently prescribed multiple medications to treat their condition, and it is important to be aware of the risk of drug-drug interactions with SSRIs; fluoxetine, fluvoxamine, and paroxetine are associated with the highest risk of CYP 450-mediated drug-drug interactions [64].

SNRI*:* By elevating both 5-HT and norepinephrine in the synaptic cleft, SNRIs are thought to alleviate depression symptoms. The reuptake of 5-HT and norepinephrine is inhibited in various ratios by the currently marketed medicines. Due to their benign safety profile, tolerance, and utility in both mental health and physical problems such as pain, SNRI drugs have been recognized as first-line therapy in the treatment of depressive disorder [61]. Richard et al. performed a randomized-controlled trial in which they concluded that extended-release venlafaxine and paroxetine were beneficial for the treatment of depression. Patients treated with either medication showed no worsening of motor function in this trial [65]. A small (n=13) open-labeled trial with a primary goal of finding the duloxetine effects on motor symptoms of PD found that it resulted in a reduction of Beck Depression Inventory (BDI) scores [66].

TCA*:* By preventing their absorption in the synaptic cleft, TCAs increase the activity of 5-HT, dopamine, and norepinephrine. TCA selectivity and potency, as well as an affinity for other receptor systems (cholinergic, histaminic, and -adrenergic), differ between drugs, resulting in varying degrees of negative effects [61]. A meta-analysis study found that antidepressant medication had a non-significant pooled impact above placebo. The combined action of the TCA (desipramine and nortriptyline) was found to have a strong antidepressant effect when these results were further examined. SSRIs (citalopram, paroxetine, and sertraline) did not have the same effect [67]. Another trial found that paroxetine had no statistically significant benefit above placebo and was inferior to nortriptyline therapy [59]. As a result of this data, the authors suggest that among patients with PD who are depressed, TCA medication may be preferred over SSRI treatment.

Despite the decreased burden of secondary amine TCA, physicians should be cautious when starting these pharmacologic medications for depression in patients with PD. Drugs should be started at the lowest effective dose and gradually increased to achieve the desired effect, keeping in mind that some patients may react to doses at the lower end of the therapeutic range [61].

MAO inhibitors*: *Due to their effect on 5-HT, norepinephrine, and dopamine, the non-selective MAO inhibitors phenelzine and tranylcypromine are used to treat depressive disorders in treatment-resistant depression (TRD). Irreversible selective monoamine oxidase B inhibitors (MAO-B), such as rasagiline and selegiline, are commonly used to treat motor symptoms of PD because of their action on dopamine [61]. Despite its label as an MAO-B inhibitor, selegiline has been shown to lose selectivity for MAO-B at doses greater than 10 mg/day, inhibiting monoamine oxidase A (MAO-A) as well. As a result, selegiline in higher doses has antidepressant characteristics [68,69]. In patients with PD who are already using antiparkinsonian and antidepressant medications, selegiline may help with both motor function and depression [69]. Safinamide inhibits MAO-B and modulates glutamate release in a highly selective and reversible manner, and it can be used as an adjuvant to L-dopa alone or in conjunction with other medications to improve 'on' time in people with PD who are in the middle to late stages of the disease [70,71].

Selective MAO inhibitors are being researched for their potential application in depression and neurodegenerative disorders [72]. Lodastigil, a dual acetylcholine-butyrylcholine esterase inhibitor, and an irreversible MAO-B inhibitor are now in phase II clinical studies for the treatment of depression and neurodegenerative illnesses such as PD [73].

Non-pharmacological Treatment

Electroconvulsive therapy (ECT)*:* ECT is usually given to patients with TRD. Borisovskaya et al. performed a systematic review and analyzed 43 studies which included one retrospective case study, two retrospective chart reviews, 27 single case reports, and 13 case series. According to the study findings, more than 93% of patients saw an improvement in their depression symptoms. Furthermore, when a motor function was reported, 83% of patients said that their symptoms had improved in addition to their depression. But these findings also revealed that there is a higher propensity of adverse effects of ECT in patients with PD. The most seen adverse effects are delirium and transient confusion, which lead to treatment discontinuation. Because autonomic dysregulation and falls are common in PD, ECT has been demonstrated to increase urine retention and fall rates potentially [74].

Cognitive-behavioral therapy (CBT)*:* As seen in the general population, achieving remission may necessitate aggressive measurement-based and stepped-care regimens that entail many pharmaceutical treatments, each lasting several months, as well as psychotherapy [75]. Although it is not always feasible to completely remove all symptoms of depression, a recovery-oriented approach highlights an individual's strengths and resilience, better function, the pursuit of goal achievement for that individual, and a sense of optimism and hope. People suffering from PD along with depression face problems such as low health literacy, access to care, availability of physicians knowledgeable about PD, and a preference for psychotherapy over drugs are all barriers to mental health treatment for depression [76].

Only two randomized-controlled clinical trials to assess the efficacy of CBT on depression in PD have been conducted to date. A randomized controlled trial conducted by Dobkin et al. found that HAM-D scores were lower in 41 patients with PD who received 10 weekly sessions of CBT + clinical monitoring (CM) compared to 39 patients with PD who received only clinical monitoring, and these scores reductions were maintained at a one-month follow up [77]. Calleo et al. conducted a pilot study in which they compared six patients allocated to usual enhanced care to 10 patients with PD via telephone delivery with their expansion of CBT services over a period of 12 weeks. This study concluded that patients with PD and depression who got CBT showed a higher reduction in depression scores from baseline than those who received usual improved care immediately after therapy and at the one-month follow-up [78].

Other Somatic Treatments

Antagonists to the N-methyl-D-aspartate (NMDA) receptor, such as MEM and ketamine, are being researched extensively to treat a variety of mental and neurological disorders. Preclinical evidence showed that the combination of MEM with antidepressants like fluoxetine and venlafaxine boosted the antidepressant impact of traditional treatments [79]. Antidepressant benefits of NMDA receptor antagonist medicines are also being investigated; ketamine has shown impressive antidepressant effects in animal trials [80]. MEM has been suggested as a potential treatment for brain illnesses such as PD, dementia, and dementia with Lewy bodies. Boxer et al. conducted a randomized, double-blinded placebo-controlled trial in the United States, which was performed over five years and published in 2013. This study included 100 subjects, of which 81 were randomized, and 76 completed the study [81]. This study concludes that patients treated with MEM had a more frequent rate of adverse events, comparable to the placebo group. MEM, according to scientists, is not an effective treatment for mild to moderate dementia with Lewy bodies.

The use of dopamines as the main treatment for PD depression has provided mixed results [82]. Pramipexole was found to be effective in lowering depressed symptoms over a 12-week period, despite minor effect sizes [83]. Fish oil, taken with or without antidepressants, improved depressive symptoms in PD patients, indicating that omega-3 consumption can be utilized as an antidepressant or as adjuvant therapy with other medications [84]. When a quick reaction is required, such as for depression-related psychosis, catatonia, or severe vegetative symptoms with impending hunger or suicidality, ECT is used. ECT is also particularly helpful in treating depressive disorders in people with PD, and it is also used to treat psychosis that has not responded to other treatments [85,86]. However, there isn't enough research to decide the potential benefits of utilizing these drugs to treat depression and neurodegenerative disorders. More research into such medicines is needed to improve the quality of life of these individuals.

The summary of included studies in this article is shown in Table 1 below.

**Table 1:** Summary of included studies regarding depression in Parkinson's disease MMSE - Mini-Mental State Examination, PDC-dAD - Provisional Diagnostic Criteria for Depression of Alzheimer's Disease, DSM - Diagnostic and Statistical Manual of Mental Disorders, GDS - Geriatric Depression Scale, UPDRS - Unified Parkinson's Disease Rating Scale, HY- Hoehn and Yahr Scale, SE - Schwab and England Scale.

REFERENCES	YEAR	DESIGN	SAMPLE SIZE	METHOD	CONCLUSION
Menon et al. [52]	2015	Prospective Cohort Study	N = 65	A total group of 45 males and 20 females with an average age of 60 years were included in the study. Patients having a score of 27 on the brief mental status evaluation were excluded from the study.	This study concluded that Not the severity of the motor symptoms or the length of the illness, but the presence of depression, is the most important predictor of low Quality of Life.
Boxer et al. [81]	2013	Randomized double-blinded Placebo trial	N = 100 (76 Completed the Study)	The required age range for the participants is 40 to 80 and they have a score of 15 on the MMSE at the time of screening.	This study concludes that Patients treated with MEM had a more frequent rate of adverse events, comparable to the placebo group.
Srivastava et al. [17]	2012		N = 247	Probands with a median age of onset of Parkinson's disease of 50 years were enlisted.	This study concluded that when compared to relatives without parkin mutations, relatives of Early-onset PD individuals with compound heterozygous mutations and no confirmed PD may have a greater risk of depression.
Belarbi et al. [16]	2010		N = 114	In the first trial, 200 people with Parkinson's disease who had been diagnosed within the previous five years were randomized. 213 people with Parkinson's disease were randomized in the second trial.	This study concluded that there is greater involvement of the limbic system in patients with LRRK2 G2019S mutation, and depression and hallucinations are more frequent among them.
Verkaik et al. [50]	2009	Cross-sectional Study	N = 518	depression diagnosis in dementia ([PDC-dAD]), dementia ([DSM], Fourth Edition-PC), and dementia stage (GDS) were all used as inclusion criteria.	This study concluded that In psychogeriatric nursing home residents with concomitant depression, irritability is one of the most common depression symptoms.
Ravina et al. [47]	2009	Double-blinded placebo-controlled trial	N = 413	The GDS 15-item Scale was used in two NIH-sponsored phase II clinical trials in Parkinson's disease, which enrolled 413 people with early, untreated PD.	This study concluded that Depressive symptoms play a significant role in disability and the choice to begin symptomatic therapy for motor-related impairment in early PD, emphasizing the necessity of recognizing and treating depression in this population.
Rojo et al. [46]	2003	Prospective Cohort Study	N = 353	UPDRS, HY, SE, MMSE, and GDS were used to assess a group of PD patients during a 9-year period.	This study concluded that a significant number of people with Parkinson's disease experience moderate to severe depression symptoms

## Conclusions

Depression is a typical nonmotor symptom of PD that can manifest during the disease, even months or years before the condition is diagnosed. The relationship between these diseases is bidirectional. Having depression increases the risk of PD and vice versa. Depression in people with PD may have a different etiology than depression in those without PD; however, this idea has not been validated, and studies thus far do not imply that it is. Standard antidepressant drugs, such as SSRI, TCA, SNRI, and MAO inhibitors, are most commonly used to treat depression in PD. Non-pharmacological interventions, such as CBT and ECT, have proven to be beneficial in the treatment of resistant depression. Finally, to provide a more coordinated and targeted strategy for diagnosing and treating depression, more research should be conducted on the relationship between PD and depression.
